# Supplementary material for: Modeling glioblastoma heterogeneity as a dynamic network of cell states
Source: Mol Syst Biol. 2021 Sep 16;17(9):e10105. doi: 10.15252/msb.202010105 (PMC8444284; doi:10.15252/msb.202010105)
Supplement: Supplementary file 6 — Source Data for Figure 5 [file MSB-17-e10105-s004.zip › Figure5A_sourcedata/GSEA_3017/nosigdb_stateE.GseaPreranked.1622795031093/RICHARDS_NOWAKOWSKI_SCIENCE_2017_EN_V1_3_UPREG.html]

Details for gene set RICHARDS\_NOWAKOWSKI\_SCIENCE\_2017\_EN\_V1\_3\_UPREG[GSEA]

|  || Dataset | state63017 |
| Phenotype | NoPhenotypeAvailable |
| Upregulated in class | na\_pos |
| GeneSet | RICHARDS\_NOWAKOWSKI\_SCIENCE\_2017\_EN\_V1\_3\_UPREG |
| Enrichment Score (ES) | 0.4446955 |
| Normalized Enrichment Score (NES) | 3.3898 |
| Nominal p-value | 0.0 |
| FDR q-value | 0.0 |
| FWER p-Value | 0.0 |
Table: GSEA Results Summary

  

Fig 1: Enrichment plot: RICHARDS\_NOWAKOWSKI\_SCIENCE\_2017\_EN\_V1\_3\_UPREG      
 Profile of the Running ES Score & Positions of GeneSet Members on the Rank Ordered List

  

| PROBE | GENE SYMBOL | GENE\_TITLE | RANK IN GENE LIST | RANK METRIC SCORE | RUNNING ES | CORE ENRICHMENT || 1 | LPL |  |  | 9 | 0.958 | 0.0201 | Yes |
| 2 | LRRN3 |  |  | 10 | 0.943 | 0.0501 | Yes |
| 3 | FABP7 |  |  | 11 | 0.920 | 0.0795 | Yes |
| 4 | PDGFA |  |  | 20 | 0.729 | 0.0934 | Yes |
| 5 | MAP1B |  |  | 22 | 0.715 | 0.1150 | Yes |
| 6 | LIMCH1 |  |  | 29 | 0.641 | 0.1285 | Yes |
| 7 | UBL3 |  |  | 53 | 0.514 | 0.1181 | Yes |
| 8 | ENC1 |  |  | 58 | 0.501 | 0.1294 | Yes |
| 9 | GAP43 |  |  | 61 | 0.494 | 0.1429 | Yes |
| 10 | DOK5 |  |  | 81 | 0.437 | 0.1347 | Yes |
| 11 | VLDLR |  |  | 87 | 0.419 | 0.1423 | Yes |
| 12 | BEX1 |  |  | 88 | 0.419 | 0.1556 | Yes |
| 13 | CHL1 |  |  | 103 | 0.392 | 0.1518 | Yes |
| 14 | ELMOD1 |  |  | 104 | 0.391 | 0.1643 | Yes |
| 15 | RTN4 |  |  | 109 | 0.388 | 0.1720 | Yes |
| 16 | EML1 |  |  | 110 | 0.383 | 0.1842 | Yes |
| 17 | MAP4 |  |  | 115 | 0.367 | 0.1913 | Yes |
| 18 | LONRF2 |  |  | 118 | 0.365 | 0.2006 | Yes |
| 19 | FAM200B |  |  | 120 | 0.361 | 0.2109 | Yes |
| 20 | GRIA1 |  |  | 122 | 0.359 | 0.2212 | Yes |
| 21 | FHL1 |  |  | 126 | 0.351 | 0.2289 | Yes |
| 22 | DSTN |  |  | 129 | 0.348 | 0.2377 | Yes |
| 23 | KHDRBS3 |  |  | 132 | 0.346 | 0.2464 | Yes |
| 24 | SHISA9 |  |  | 133 | 0.345 | 0.2574 | Yes |
| 25 | TCEAL4 |  |  | 135 | 0.344 | 0.2672 | Yes |
| 26 | NUDT3 |  |  | 136 | 0.343 | 0.2781 | Yes |
| 27 | DAAM1 |  |  | 138 | 0.343 | 0.2879 | Yes |
| 28 | BNIP3L |  |  | 142 | 0.341 | 0.2953 | Yes |
| 29 | PRMT2 |  |  | 152 | 0.333 | 0.2954 | Yes |
| 30 | WWC1 |  |  | 154 | 0.329 | 0.3048 | Yes |
| 31 | TUBB2A |  |  | 157 | 0.327 | 0.3129 | Yes |
| 32 | DPYSL3 |  |  | 161 | 0.325 | 0.3197 | Yes |
| 33 | ATXN1 |  |  | 174 | 0.315 | 0.3158 | Yes |
| 34 | NLGN4X |  |  | 177 | 0.312 | 0.3234 | Yes |
| 35 | GAS7 |  |  | 180 | 0.310 | 0.3310 | Yes |
| 36 | CALM2 |  |  | 191 | 0.303 | 0.3290 | Yes |
| 37 | SPTBN1 |  |  | 194 | 0.300 | 0.3363 | Yes |
| 38 | SDC3 |  |  | 200 | 0.295 | 0.3399 | Yes |
| 39 | DKK3 |  |  | 205 | 0.293 | 0.3445 | Yes |
| 40 | ACSL3 |  |  | 207 | 0.291 | 0.3526 | Yes |
| 41 | KIFAP3 |  |  | 208 | 0.290 | 0.3619 | Yes |
| 42 | NLN |  |  | 214 | 0.286 | 0.3652 | Yes |
| 43 | FERMT2 |  |  | 219 | 0.283 | 0.3696 | Yes |
| 44 | SCG2 |  |  | 220 | 0.283 | 0.3786 | Yes |
| 45 | SRGAP1 |  |  | 223 | 0.281 | 0.3852 | Yes |
| 46 | CXADR |  |  | 232 | 0.275 | 0.3847 | Yes |
| 47 | CHMP2A |  |  | 243 | 0.266 | 0.3815 | Yes |
| 48 | IAH1 |  |  | 244 | 0.265 | 0.3900 | Yes |
| 49 | CSRP2 |  |  | 247 | 0.264 | 0.3961 | Yes |
| 50 | HMGCR |  |  | 251 | 0.261 | 0.4009 | Yes |
| 51 | PGM2L1 |  |  | 255 | 0.260 | 0.4057 | Yes |
| 52 | GSTM3 |  |  | 257 | 0.259 | 0.4128 | Yes |
| 53 | ATP9A |  |  | 264 | 0.255 | 0.4139 | Yes |
| 54 | TMEM245 |  |  | 272 | -0.250 | 0.4138 | Yes |
| 55 | TNPO2 |  |  | 273 | -0.251 | 0.4218 | Yes |
| 56 | SUPT16H |  |  | 276 | -0.251 | 0.4275 | Yes |
| 57 | MICU1 |  |  | 279 | -0.253 | 0.4332 | Yes |
| 58 | TUBA1C |  |  | 282 | -0.253 | 0.4389 | Yes |
| 59 | DRAXIN |  |  | 285 | -0.254 | 0.4447 | Yes |
| 60 | FAT3 |  |  | 311 | -0.261 | 0.4239 | No |
| 61 | RAD51C |  |  | 333 | -0.264 | 0.4080 | No |
| 62 | CUEDC2 |  |  | 335 | -0.265 | 0.4152 | No |
| 63 | SLK |  |  | 343 | -0.266 | 0.4156 | No |
| 64 | ADCY1 |  |  | 347 | -0.268 | 0.4207 | No |
| 65 | PGD |  |  | 355 | -0.270 | 0.4211 | No |
| 66 | RNF165 |  |  | 382 | -0.276 | 0.3997 | No |
| 67 | HSPA8 |  |  | 392 | -0.280 | 0.3981 | No |
| 68 | CDC123 |  |  | 394 | -0.280 | 0.4059 | No |
| 69 | GDI2 |  |  | 401 | -0.283 | 0.4080 | No |
| 70 | SLIT1 |  |  | 408 | -0.285 | 0.4101 | No |
| 71 | PPA1 |  |  | 420 | -0.289 | 0.4065 | No |
| 72 | SATB2 |  |  | 429 | -0.292 | 0.4065 | No |
| 73 | TTL |  |  | 440 | -0.297 | 0.4043 | No |
| 74 | HOMER1 |  |  | 463 | -0.304 | 0.3884 | No |
| 75 | VDAC2 |  |  | 492 | -0.311 | 0.3658 | No |
| 76 | KLHL4 |  |  | 496 | -0.313 | 0.3722 | No |
| 77 | PA2G4 |  |  | 501 | -0.314 | 0.3776 | No |
| 78 | TAGLN3 |  |  | 522 | -0.321 | 0.3646 | No |
| 79 | TRIO |  |  | 525 | -0.322 | 0.3725 | No |
| 80 | C2orf69 |  |  | 556 | -0.338 | 0.3484 | No |
| 81 | TSPAN14 |  |  | 581 | -0.350 | 0.3316 | No |
| 82 | GGCT |  |  | 598 | -0.356 | 0.3244 | No |
| 83 | SEZ6L |  |  | 642 | -0.383 | 0.2866 | No |
| 84 | RNF157 |  |  | 669 | -0.409 | 0.2694 | No |
| 85 | TP53I11 |  |  | 718 | -0.445 | 0.2278 | No |
| 86 | STMN1 |  |  | 778 | -0.529 | 0.1760 | No |
| 87 | TUBB |  |  | 802 | -0.570 | 0.1674 | No |
Table: GSEA details [plain text format]

  

Fig 2: RICHARDS\_NOWAKOWSKI\_SCIENCE\_2017\_EN\_V1\_3\_UPREG: Random ES distribution      
 Gene set null distribution of ES for **RICHARDS\_NOWAKOWSKI\_SCIENCE\_2017\_EN\_V1\_3\_UPREG**
